# Supplementary material for: Tumor Necrosis Factor Receptor 2 Inhibits HIV-1 Infection by Blocking the Binding of gp120 to CD4
Source: Int J Biol Sci. 2026 Apr 8;22(8):4303–14. doi: 10.7150/ijbs.124330 (PMC13137973; doi:10.7150/ijbs.124330)
Supplement: Supplementary file 1 — Supplementary figures. [file ijbsv22p4303s1.pdf]

Supplementary Figure 1

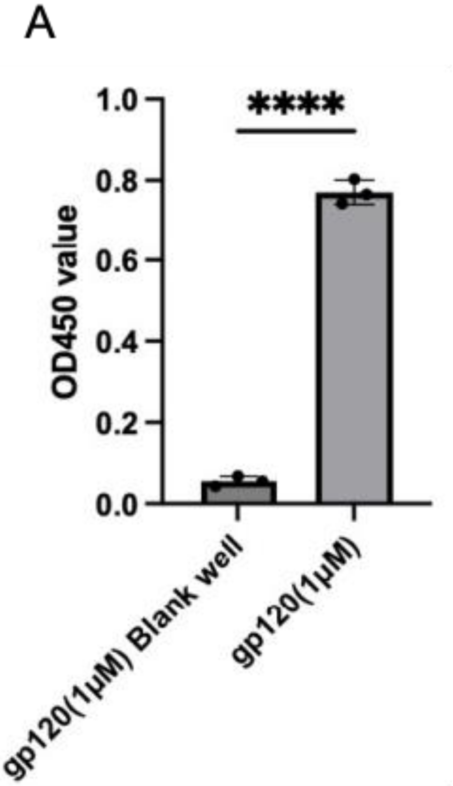

Supplementary Figure 1 (A) As a negative control, gp120 was added to uncoated (blank) wells, and no significant signal was observed, confirming minimal non-specific binding. One-way ANOVA was used to analyze group differences. Significance levels: * $p < 0.05$, ** $p < 0.01$, *** $p < 0.001$, **** $p < 0.0001$, ns no significant difference.

Supplementary Figure 2

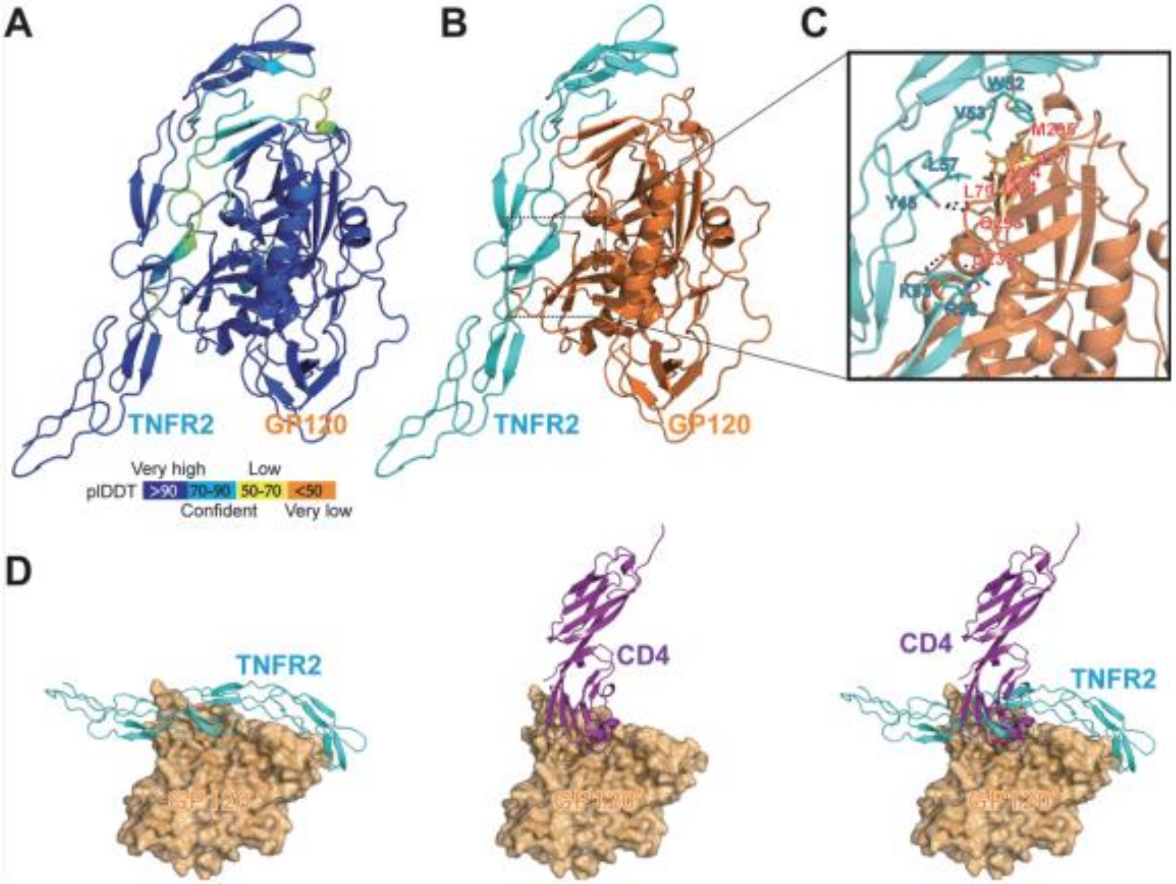

Supplementary Figure 2 The TNFR2 epitope on gp120 aligns well with the CD4 binding epitope. **(A)** Evaluation of TNFR2–gp120 binding site interactions of Alpha Fold 3 structures. pLDDT as a measure of conformational plasticity. **(B)** The interactions of TNFR2 (blue) and gp120(orange). **(C)** Hydrogen bonds between residues in the TNFR2-gp120 complexes. **(D)** Comparison of the binding epitopes of TNFR2 and CD4 on gp120.

Supplementary Figure 3

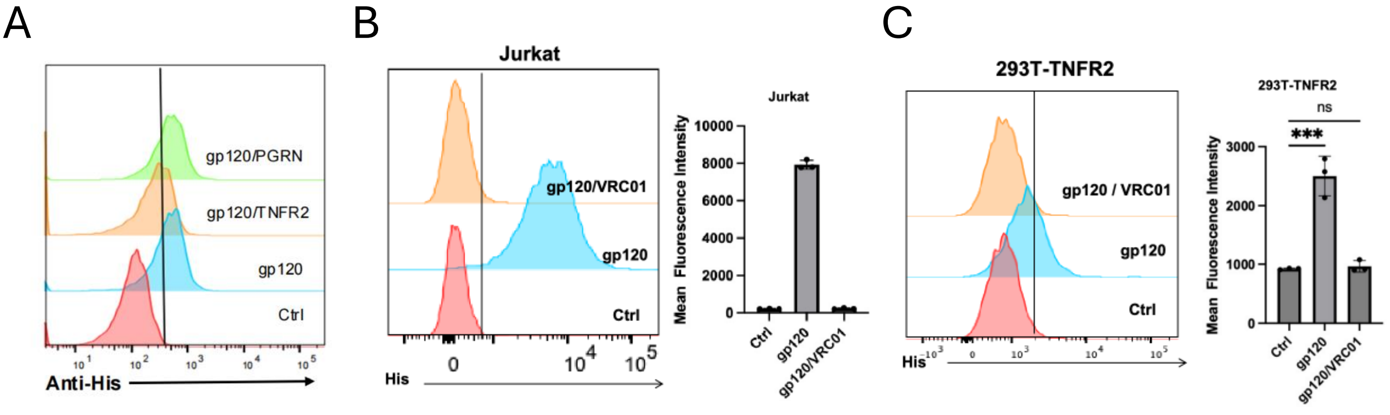

Supplementary Figure 3 (A) Flow cytometry was utilized to evaluate cell-bound His-tag, indicating the binding of gp120-His to the Jurkat cells, and also assessed if TNFR2 (1 μM) or no related control protein progranulin (PGRN) can block gp120-cell binding. The statistical analysis of the double positive cells. **(B)** VRC01 blocks gp120 binding to TNFR2 by targeting the CD4 binding site. Jurkat T cells were incubated with gp120 protein in the presence or absence of the VRC01 antibody. Binding of gp120 to CD4 was assessed by flow cytometry using an anti-His tag antibody to detect surface-bound gp120. **(C)** 293T cells overexpressing human TNFR2 were used to evaluate the effect of VRC01 on gp120–TNFR2 interaction. Pre-incubation with VRC01 reduced gp120 binding to TNFR2, as measured by His tag detection via flow cytometry. One-way ANOVA was used to analyze group differences. Significance levels: * $p < 0.05$, ** $p < 0.01$, *** $p < 0.001$, **** $p < 0.0001$, ns no significant difference.

Supplementary Figure 4

A

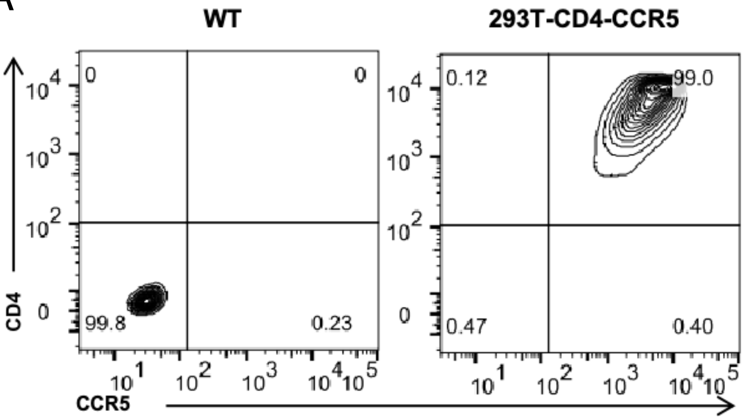

Supplementary Figure 4 (A) Flow cytometry was performed to evaluate the surface expression levels of CD4 and CCR5 on 293T-CD4-CCR5 cells.
